# Application of Bulk-Fill Composite to Simplify the Cementation of Indirect Restorations: The COMBO Technique

**DOI:** 10.3390/dj12080239

**Published:** 2024-07-29

**Authors:** Giuseppe Chiodera, Riccardo Monterubbianesi, Vincenzo Tosco, Ombretta Papini, Giovanna Orsini, Angelo Putignano

**Affiliations:** 1 Private Practice, 25123 Brescia, Italy; chiuseppe@gmail.com (G.C.); ombry@hotmail.com (O.P.); 2 Department of Clinical Sciences and Stomatology, Università Politecnica delle Marche, 60126 Ancona, Italy; v.tosco@pm.univpm.it (V.T.); g.orsini@staff.univpm.it (G.O.); a.putignano@staff.univpm.it (A.P.); 3 National Institute of Health and Science of Aging (INRCA), 60124 Ancona, Italy

**Keywords:** luting agents, luting cement, bulk-fill composite, indirect restoration, luting technique, cementation technique

## Abstract

This article proposes a technique to simplify the cementation of indirect restorations by exploiting the advantageous properties of bulk-fill composites (BFCs). The proposed technique consists of using a thin layer of a high-viscosity (HV) BFC in the interproximal margins of the preparation and applying low-viscosity (LV) resin luting agents (RLAs) to the rest of the prepared surface. The application of the HV BFC limits the extrusion of the LV RLAs in the interproximal area, deviating the excesses of LV RLAs only on the vestibular and lingual side. This deviation allows the management and control of the excess material in complicated interproximal spaces, simplifying the cementation procedure of indirect restorations and achieving a reliable final result in terms of removing excess in a safe and repeatable way. This technical report provides an alternative clinical approach for cementing indirect restorations using the consistency and viscosity of different RLAs.

## 1. Introduction

The development of new resin-based materials in adhesive dentistry has changed the protocols for the preparation and cementation of indirect restorations. Indeed, conservative tooth preparation and resin-based luting agents (RLAs) are becoming the gold standard in indirect restorations [1,2]. Since these restorations can strongly adhere to the residual dental tissues using RLAs, a clinician can preserve as much healthy tissue as possible and perform a non-retentive preparation [3].

Most RLAs have a low viscosity (LV) and can be divided into several main families based on the material (resin cements and resin-based composites), type of activation (light or dual-cured), and adhesive procedures (self-adhesive or not self-adhesive) [4]. Although each family of RLAs possesses its own advantages and disadvantages in terms of chemical, aesthetic and physical properties, their consistency influences the management of material excesses at the preparation margin, mainly in the interproximal area where their removal could be difficult. Even today, during the cementation of an indirect restoration, excess removal remains a crucial step for the survival rate of the restoration, particularly in those cases in which such residuals are close to the gingival margin [5]. Indeed, at the gingival level, rough and irregular surfaces can increase plaque accumulation and, hence, periodontal issues.

In addition to the LV RLAs, a high-viscosity (HV) resin material was investigated for cementing indirect restorations, obtaining promising results in terms of its physical and adhesive properties [6]. In particular, several authors have proposed preheating or ultrasonically activation HV RLAs to improve the handling and chemical properties of HV RLAs, simplifying the removal of the excess [7,8]. Nevertheless, these procedures present limits correlated to the gradual heat loss over time [9]. Indeed, 15 s after the end of heating, HV RLAs had an average temperature below 50 °C, with temperature losses varying between 45% and 61% [9]. Since the consistency and handling of thermally or sonically activated HV RLAs are time-dependent, such materials should be used quickly and efficiently, requiring clinician-specific training [10]. These materials are widely used in clinical practice with different application techniques; however, there is no consensus in the scientific literature regarding which RLA or cementation technique is the best for cementing indirect restorations. 

Considering the issues associated with LV RLAs and HV pre-heated and ultrasonically activated RLAs, this case report proposes the possibility of using a bulk-fill composite (BFC) for the cementation of an indirect restoration. BFCs are mainly used for direct restorations, allowing an adequate polymerization of a 4 mm-thick layer of material. The availability of both LV and HV BFCs allows clinicians to choose the most suitable material in relation to the clinical situation [11]. LV BFC offers the advantages of its fluid consistency during application in situations with a complex cavity design. HV BFC allows the whole cavity to fill without any additional occlusal coverage [12]. In addition, all these BFCs possess advantageous properties such as high physical and chemical properties, lower polymerization shrinkage and stress and reduced cusp deflection. 

For these reasons, the proposed technique, called COMBO, combines the main properties of LV RLAs together with the HV BFC to facilitate the cementation of indirect restorations, which has always been a challenge for clinicians. This article provides a step-by-step description of the COMBO technique through a case report and can be considered as inspiration for further in vivo and in vitro investigations related to this topic. Indeed, this case report is the first to describe a new cementation technique for indirect restorations using BFCs.

## 2. Case Report

**Figure 1:**
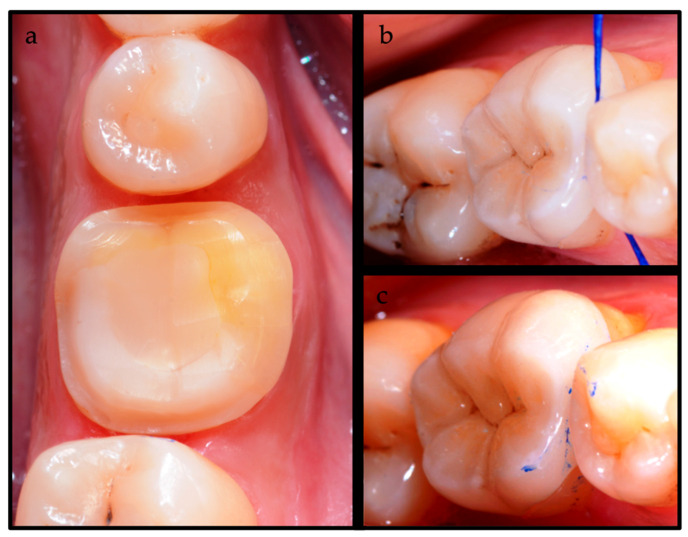
(**a**) Occlusal view of the indirect preparation (the mandibular first molar, previously endodontically treated) after removal of provisional restoration and cleaning of the surface; (**b**,**c**) control of contact points by flossing marked with articulated paper.

A 54-year-old female patient presented themselves to a private practice office for an emergency regarding her first mandibular molar. After the diagnosis of irreversible pulpitis, different treatment options were considered, and once they were all explained in detail, the patient accepted the proposed treatment plan, signing an informed consent form. The treatment plan consisted of an endodontic treatment followed by an indirect restoration. In addition, the patient was aware of the COMBO protocol for cementing the indirect restoration. Since the focus of this case report concerns the description of the cementation technique, the clinical phases of the endodontic treatment [13] will be omitted, starting the case description directly at the cementation appointment. Firstly, a trick for facilitating the final step was to check the normal occlusion before the indirect restoration try-in (Figure 1). When possible, as it was in this case, local anesthesia was avoided in order to obtain a reliable check of the occlusion without altering the patient’s perception. During the try-in of the indirect restoration, a floss marked by an articulating paper of 40 μm (TrollFoil Blue, TrollDental USA Inc., Upplands Väsby, Sweden) was used in the interproximal contact point for checking the proper contact points. Instead, gaps between the preparation and the indirect restoration were evaluated using a pointed instrument. 

**Figure 2:**
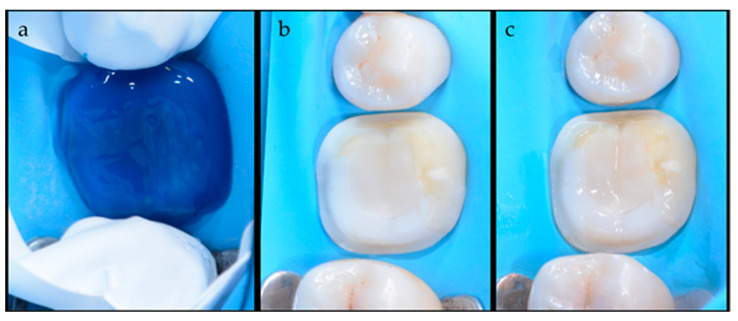
(**a**) The etching step is performed with 37% phosphoric acid under a rubber dam. Matrices or PTFE tapes can be used to achieve adequate isolation and protect neighboring teeth from being over-etched.; (**b**) the typical opaque aspect of the etched surfaces; (**c**) the prepared tooth after adhesive application.

After all these procedures, the intaglio surface was cleaned using an ultrasonic bath. A rubber dam was used to isolate the teeth and the adaptation of the indirect restoration was checked again. Then, the prepared tooth surface was cleaned and conditioned using an air-abrasion machine (AquaCare, Akura Medical, Los Gatos, CA, USA), the surface was rinsed thoroughly and dried. In the meantime, the adjacent unprepared teeth were protected using polytetra-fluoroethylene (PTFE) tape from etching (Figure 2). After the adhesive procedures on the dental substrates and the air-blowing, the bonding agent was cured following the manufacturer’s instruction. Regarding the preparation of the indirect restoration, the intaglio surface was treated according to the material and the bonding was applied on the intaglio surface and light-cured [14]. The indirect restoration, made out of lithium disilicate [15] (IPS e-max Press, Ivoclar Vivadent, Schaan, Liechtenstein), was seated and pushed gently along the insertion axis until it was fully seated.

**Figure 3:**
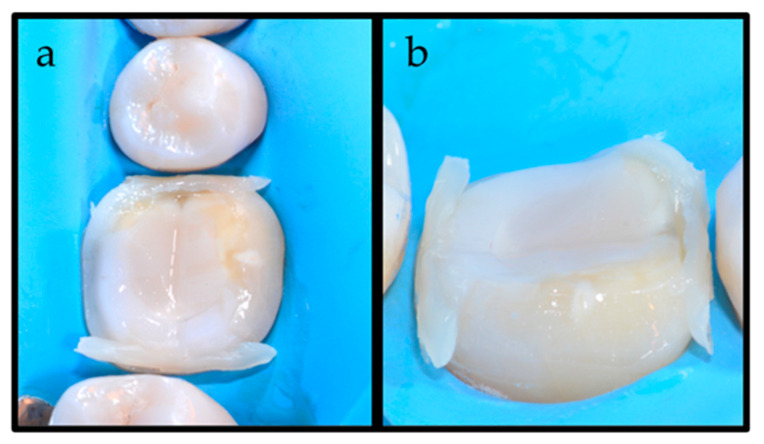
(**a**) Two little pieces of a “spaghetti-like” high-viscosity bulk-fill composite have been applied on the interproximal margins; (**b**) a vestibular view of the “spaghetti-like” application.

**Figure 4:**
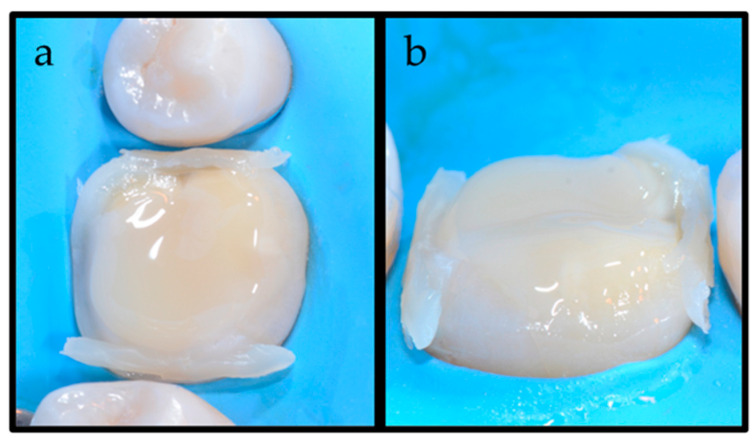
(**a**) The application of a low-viscosity (LV) luting agent on the prepared tooth after placement of the “spaghetti-like” high-viscosity bulk-fill composite; (**b**) a vestibular view of the combination of high- and low-viscosity luting cements.

At this point, the COMBO technique was applied. Two little pieces of a “spaghetti-like” HV BFC (Filtek One Bulk Fill Restorative, 3M ESPE, St. Paul, MN, USA) were placed on the interproximal side of the preparation margins (Figure 3), while the rest of the preparation was filled by LV RLAs (Filtek Bulk Fill Flow, 3M ESPE, St. Paul, MN, USA) (Figure 4). 

**Figure 5:**
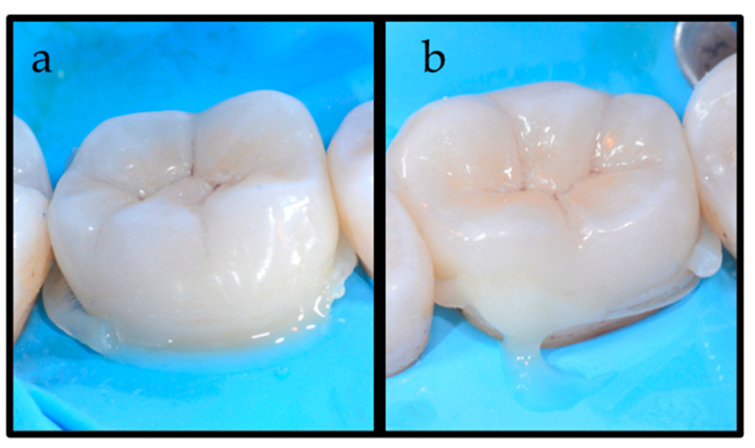
(**a**) Vestibular and (**b**) lingual view of the diffusion of the luting agents after placement of the indirect restoration. The difference in viscosity of the luting agents is evident: the LV luting agent is observed in the lingual area, while HV BFC is present in the interproximal areas.

**Figure 6:**
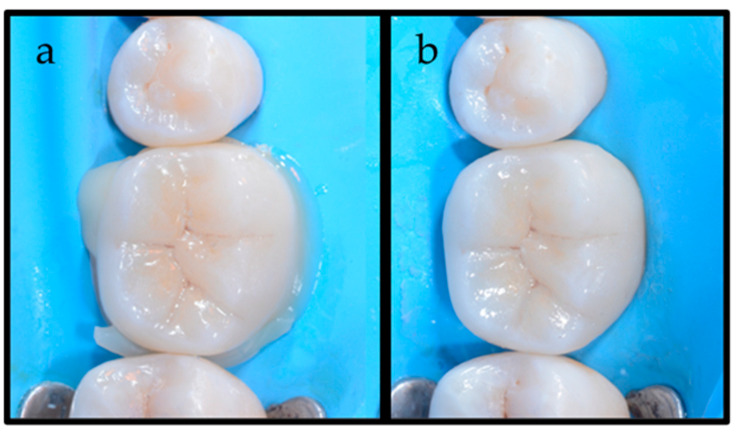
(**a**) Occlusal view of the lithium disilicate indirect restoration in place before excess removal; (**b**) Occlusal view after the excess removals.

**Figure 7:**
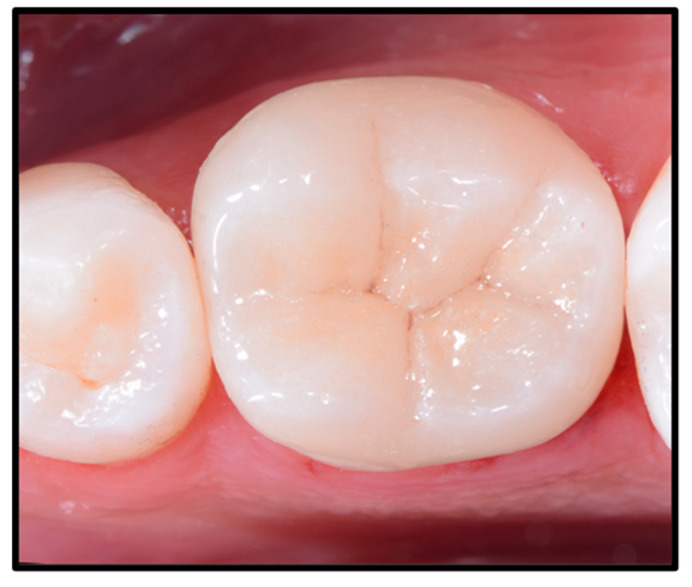
Occlusal view after rubber dam removal and after the finishing and polishing procedures.

**Figure 8:**
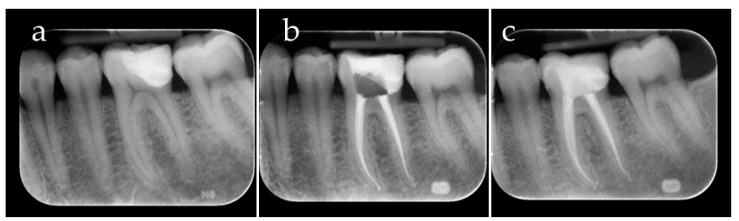
(**a**) A pre-operative radiograph of the first mandibular molar; (**b**) a radiograph after the endodontic treatment; (**c**) a post-operative radiograph after the indirect restoration cementation.

In this phase, the COMBO technique allowed the excesses of the LV material to deviate only on the vestibular and lingual side of the preparation; instead, on the interproximal area, few excesses of HV BFC were removed (Figure 5). Excess cement from the buccal and lingual/palatal surfaces was gently removed with a microbrush, while the excess from the interproximal area was removed with a fine-pointed instrument (LM-Arte Fissura, LM-Instruments Oy, Parainen, Finland). Subsequently, a 5 s pre-polymerization allowed the luting agents to harden, facilitating the removal of further excesses [16,17]. To ensure that the resin material was cured properly, glycerine was applied in the margins to exclude oxygen inhibition, and 60 s of polymerization occurred on the mesio-buccal, disto-buccal, mesio-lingual, disto-lingual and occlusal surfaces. Polymerization was carried out by means of the curing lamp LED Elipar (Elipar S10, 3M ESPE, St. Paul, MN, USA) with a light irradiance of 1200 mW/cm^2^ (Figure 6). After rubber dam removal, margins were finished and polished (Figure 7) [18]. Finally, the adaptation and the fit of the indirect restoration was checked through a dental radiographic examination (Figure 8). 

## 3. Discussion

In recent years, a wide variety of materials with superior and high-performing characteristics have been proposed for the cementation of indirect restorations. Although dual-cured and light-cured RLAs can simplify the cementation procedure, their application is influenced by the distance from the tip of the curing lamp to the margin preparation and the consistency and viscosity of the RLA. However, when possible, clinicians prefer light-cured RLAs instead of dual-cured RLAs in thin indirect restorations because they may present unpleasant color changes over time, causing aesthetic problems in the preparation margin [19]. Furthermore, a dual-cured RLA hardens in a short amount of time, making the removal of excesses a sensitive procedure that requires speed in execution, and hence, the operators are required to have great skill and experience. If excesses remain, they can provoke an irregularity on the preparation margin, which can increase the risk of discolorations, secondary caries [20] and periodontal issues. Indeed, Sirajuddin et al. highlighted the detrimental impact of the residual RLA on the periodontal tissue, resulting in the accumulation of plaque and oral pathogens, which can lead to periodontal disease [21]. Lee et al. found that undetected RLA remnant excesses were more likely to be found when the restorations were placed in deeper subgingival margins [22]. In this light, the complete removal of RLA from peri and subgingival areas is fundamental, especially when the indirect restoration preparation margins are in the proximity of the cementum enamel junction. Regarding the consistency and viscosity of RLAs, although the LV RLA makes the fitting of indirect restorations easier than the HV RLA, it tends to also accumulate material excesses on the interproximal area. Moreover, one of the attempts to solve the management of material excess and increase the mechanical properties of LV RLAs was the introduction of the heating and preheating of an HV resin-based composite [10]. Indeed, it is possible to use a light-cured HV resin-based composite after its heated activation for the cementation of an indirect restoration. However, even though many studies have addressed the performance of different materials with preheating techniques, there is a lack of evidence that preheating a resin-based composite improves the quality and durability of indirect restorations [9]. Moreover, preheating the material could increase the temperature of the pulp chamber, and a variation of 5.5 °C is considered harmful to human pulp tissue [23]. In addition, the use of a preheated resin-based composite as an RLA negatively influences the adaptation of indirect restorations, due to several variables such as the formulation of the material or the heating time and temperature, showing the high heterogeneity of the results [9]. 

For these reasons, this technical report aims to propose a procedure using a BFC that allows excesses to be removed in a simple and more predictable way, without preheating the resin-based composite. Indeed, the concept behind the COMBO technique consists of applying an HV BFC in the interproximal margins of the preparation to guide the excess of the LV RLA towards the vestibular and lingual/palatal sides, as the removal procedures are easier to perform there than on the interproximal sides, regardless of the working time and the clinician’s expertise.

The advantages of using a BFC instead of a traditional resin-based composite lies in the fact that a proper polymerization can be reached even at 4 mm of distance from the tip of the curing lamp, followed by high bond strength values. Studies reported that the viscosity of BFCs does not influence their microhardness [24] and capability to bond dentine, which are similar to those of conventional resin-based composites [25]. In addition, since a BFC contains more fillers than a dual-cured RLA, it presents superior intrinsic mechanical properties [25]. Moreover, the higher filler content of a BFC and the lower initiator concentration compared with those that are dual-cured might be advantageous in terms of mechanical strength and the wear properties of the exposed margins [26]. Although some authors reported that using HV composites for luting ceramic partial crowns causes marginal misfitting, they did not consider the combination of different consistencies [27]. 

Although choosing the right RLA and managing excesses are fundamental steps, the survival rate of the indirect restoration also depends on the proper fitting of the indirect restoration with the proper contact area. In this light, the COMBO technique proposes two different checkpoints. The first one involves the use of a marked dental floss to control the proper contact point of the indirect restoration before the cementation, since a tight contact point can influence the adaptation of the indirect restoration. The second crucial checkpoint consists of the evaluation of the interproximal margin using a dental x-ray examination. Indeed, although an X-ray provides a 2D image, it represents an important tool to evaluate the excesses remaining in the interproximal area, which surely represents the critical area of the luting procedure. Therefore, the COMBO technique may provide a trick for simplifying the cementation procedures of an indirect restoration, especially in the case of a partial crown overlay, deep margins and tight interproximal areas. Although the stability over time of the individual HV BFC and LV RLA has already been studied [28,29], the limitation of this study concerns the lack of data on the physical and mechanical properties of their combinations. It is noteworthy that the heated resin composites were not included in this technique since, in addition to the above-mentioned reasons, a recent review stated that they negatively influence the adaptation of fixed dental prostheses [9]. 

The use of COMBO could be a feasible and repeatable technique to cement indirect restorations; however, the authors suggest checking the viscosity of resin materials before using them directly on the patient. Then, the main clinical implication of this technique is the easier removal of excesses by using a common material such as a BFC, although further in vitro and in vivo studies are needed to validate its stability over time. Indeed, the limitation of this technique is that there are no studies in the literature on this topic. 

## 4. Conclusions

In conclusion, the proposed COMBO technique can serve as an alternative clinical approach for the cementation of indirect restorations, simplifying the removal of excess material through the application of different viscosities of materials. 

Furthermore, the COMBO technique is suitable for use by both experienced and inexperienced clinicians, as the use of light-curing resins, such as BFCs, provides the operator with sufficient time to work with the material on the preparation margins. Moreover, clinicians can employ various combinations of materials and brands for the COMBO technique, if the fundamental principle of varying material viscosity is respected.

## Data Availability

The STL images used for this study are available from corresponding author on reasonable request.
